# Analysis and classification of the road traffic health and safety mobile apps based on the Haddon’s matrix 

**Published:** 2019-08

**Authors:** Hossain Aghayari, Leila R Kalankesh, Homayoun Sadeghi Bazargani

**Affiliations:** ^*a*^Department of Health Information Technology, School of Management and Medical Informatics, Tabriz University of Medical Sciences, Tabriz, Iran.; ^*b*^Health Services Management Research Center, Research Center of Clinical Psychiatry and Behavioral Sciences, and School of Management and Medical Informatics, Tabriz University of Medical Sciences, Tabriz, Iran.; ^*c*^Road Traffic Injury Research Center, Tabriz University of Medical Sciences, Tabriz, Iran.

## Abstract

**Background:**

Road traffic accidents have been one of the main causes of death worldwide. However, most of the crashes are both predictable and preventable. With the widespread use, mobile phone is considered as a major road safety risk. However, varieties of apps have recently been developed for improving the road traffic health and safety. Despite an increasing trend of these apps, there is no comprehensive analysis of their features and no taxonomy or classification of them based on the traffic safety theories or frameworks. The aim of this study was to explore characteristics of available mobile apps on the road traffic health and safety and classify them based on the Haddon’s Matrix.

**Methods:**

To conduct this research, a comprehensive and systematic review of the mobile applications developed for the road traffic health and safety was carried out through using the qualitative content analysis. Google Play was searched through using a combination of the keywords to retrieve the road traffic apps. In order to extract the app features, their description was examined, and their content was analyzed. Then they were classified in four main categories including Road Traffic Health & safety, Road Traffic Training, Road traffic Navigation, and Other Road Traffic apps. Finally, the Haddon’s matrix was applied to analyze and classify those mobile apps residing in two main categories of the traffic health & safety, and the traffic training. Haddon’s matrix is a relevant framework for structured analyses of traffic injury events. In the Haddon’s matrix, the contributions of human, vehicle/equipment and environmental factors to the injuries in the three phases pre-crash, crash and post-crash has been presented.

**Results:**

In this study, 916 mobile apps met the inclusion criteria and were included in the final analysis.19 subcategories were identified for classifying the included apps based on their features and functionalities. In total, 620 of the mobile apps were grouped on the basis of Haddon’s matrix. About 61.76% of these apps were categorized as the traffic health & safety group. Among the subcategories of the traffic health & safety group, the highest number of apps (194) was the real-time traffic alerting apps. Behavior based feed-backing subcategory was ranked the second among the traffic health and safety apps with 75 apps.

When the researchers applied factors and phases of Haddon’s framework for analyzing the apps, it was found that the Haddon’s factors have been taken into account in the apps grouped into two main categories of the apps including traffic training and traffic health & safety applications. The highest percentage of Haddon’s matrix factor considered for the intervention through the apps was related to the physical & social environment (23.69%) in the traffic training category. The features of 259 apps were classified in the Event/ Driving phase of Haddon’s matrix. Most of the apps in this phase was related to the real-time traffic alerting subgroup. These apps performed in real time while driving. The lowest number of the apps in driving phase was observed in the behavior based feed backing subcategory, which inform drivers about their driving behavior in real-time. Sixteen apps had features that categorized them in both Pre-event / Pre-driving and Event / Driving phases. In addition, 20 apps were found to have feature for intervening in both Event / Driving and Post-event / Post –driving phases. All 235 apps in the traffic training category were in pre-event/ pre-driving phase, which requires users to use these apps before driving.

**Conclusions:**

Applying Haddon’s matrix on analyzing and classifying the traffic health and safety apps revealed strengths and weaknesses of the existing related mobile apps in terms of the factors that must be considered for intervention toward prevention and reduction of road traffic accidents as the main public health issue.

**Keywords:**

Mobile apps, Traffic accident prevention apps, Traffic safety, Haddon matrix, Public health

